# Evidence of the Possible Interaction between Ultrasound and Thiol Precursors

**DOI:** 10.3390/foods9010104

**Published:** 2020-01-19

**Authors:** Tomas Roman, Loris Tonidandel, Giorgio Nicolini, Elisabetta Bellantuono, Laura Barp, Roberto Larcher, Emilio Celotti

**Affiliations:** 1 Fondazione Edmund Mach—Technology Transfer Center, via Edmund Mach 1, 38050 San Michele all’Adige, Italy; 2 Department of Agricultural, Food, Environmental and Animal Sciences, University of Udine, Via Sondrio 2/A, 33100 Udine, Italy

**Keywords:** ultrasound, 3-mercaptohexan-1-ol, 4-mercapto-4-methylpentan-2-one, thiol precursors, grape juice, maceration

## Abstract

The effect of ultrasound (20 kHz, 153 μm) on the prefermentation extraction mechanisms in Sauvignon Blanc grapes was studied, focusing on 3-mercaptohexan-1-ol (3MH) and 4-mercapto-4-methyl-pentan-2-one (4MMP) precursors linked to glutathione (GSH) and cysteine (Cys). The treatment determined a positive extraction trend between the duration (untreated, 3 and 5 min) and the conductivity or the concentration of catechins and total phenols, significantly differentiated after 5 min. Nevertheless, the concentration of the thiol precursors in grape juice not only remained undifferentiated, but that of 3-S-glutathionyl mercaptohexan-1-ol showed a negative trend with the treatment time applied (168 ± 43, 156 ± 36, and 149 ± 32 μg/L, respectively, for control, 3 and 5 min). The divergence on the effect between families of compounds suggests an interaction between the sonication treatment and thiol precursor molecules. In order to evaluate the possible degradation properly, ultrasound was applied in a model solution spiked with 3MH and 4MMP precursors, reproducing the conditions of grapes. Except for Cys-3MH, the mean concentration (*n* = 5) for the rest of the precursors was significantly lower in treated samples, predominantly in those linked to glutathione (~−22% and ~18% for GSH-3MH and GSH-4MMP) rather than to cysteine (~−6%~−8% for Cys-3MH and Cys-4MMP). The degradation of precursors was associated with a significant increase of 3MH and 4MMP. The formation of volatile thiols following sonication is interesting from a technological point of view, as they are key aroma compounds of wine and potentially exploitable in the wine industry through specific vinification protocols.

## 1. Introduction

**Figure 1:**
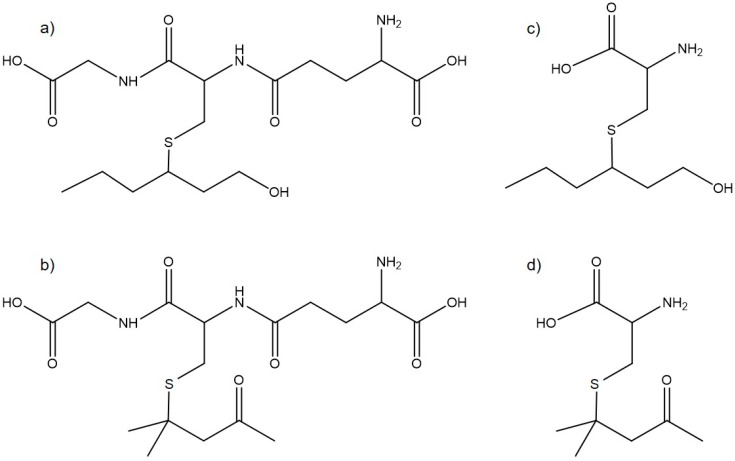
Chemical structure of thiol precursors. (**a**) 3-S-glutathionyl mercaptohexan-1-ol (GSH-3MH), (**b**) 3-S-glutathionyl mercapto-4-methyl-pentan-2-one (GSH-4MMP), (**c**) 3-S-cysteinyl mercaptohexan-1-ol (Cys-3MH), and (**d**) 3-S-cysteinyl mercapto-4-methyl-pentan-2-one (Cys-4MMP).

Thiols are molecules featuring a carbon bond to a sulfydryl group. The so-called polyfunctional thiols represent an important subclass in winemaking and juice production because of their extremely high aromatic power. Among polyfunctional thiols, 4-mercapto-4-methylpentan-2-one (4MMP), 3-mercaptohexan-1-ol (3MH), and 3-mercaptohexil acetate (3MHA) should be highlighted as the compounds with the greatest impact, due to their concentration in wine, odour activity values, and pleasing aroma [1]. Thiol scents evoke grapefruit, black currant or passion fruit, although these cannot be usually detected during grape tasting. The odoriferous molecules are present in grape berries as nonvolatile precursors (Figure 1) and are linked to some amino acids or di- and tri- peptides, e.g., cysteine or glutathione, through a sulfur bond [1].

The precursor is created for detoxification following the production of hexen-1-al as a plant response to stress, using glutathione as the original chelating molecule [2]. The adduct is then transported and accumulated into the vacuoles [3], mainly of the skin cells [4]. During grape crushing and pressing, some of them are released into the must, with concentrations of several mg/L [5] and a final level highly dependent on the environmental and technological conditions [6,7] in addition to the grape cultivar [8]. Marc is also reported to show a significant content after pressing [9] even if their content at industrial conditions is not completely studied. The remaining precursors could be then considered as a loss in the aroma potential of wines. The release of 4MMP or 3MH from grape precursors occurs during the alcoholic fermentation because of the cleavage of the S- bond by means of yeast-strain-dependent specific β-lyases [10]. However, it is not the only pathway, and the direct formation of free molecules during alcoholic fermentation has been also reported [11].

In order to enhance the extraction yield of aroma compounds, different winemaking techniques can be applied, such as prefermentation skin contact maceration. This operation normally needs time, which increases the risk of development of spoilage microorganisms, as well as energy to chill the must in order to keep the microbes under control. Recently, the International Organization of Vine and Wine has approved the use of a new technology to increase extraction from grape tissues during winemaking based on sonication [12]. Ultrasound (US) consists of mechanical sound waves with frequencies ranging from 20 kHz to 10 MHz. Depending on the frequency and the energy applied, they can be divided into high frequency ultrasound (100 kHz–1 MHz) and power ultrasound (20–100 kHz). This differentiation makes it possible for ultrasound to be used in the food industry, as power US can interact with matter [12]. US causes the creation of multiple series of compression and rarefaction that can lead to acoustic cavitation in a liquid medium, producing micro-bubbles (small amounts of steam). The violent collapse of these bubbles generates micro-zones with extremely high temperature conditions, according to the hot-spot theory that predicts temperatures of many thousands of degrees of Kelvin and pressures of hundreds of MPa inside the bubbles during the compression cycle [13]. This activates ionisation mechanisms and forms radicals in the gas phase, which in the liquid, can undergo redox reactions. The hot interfacial region created between liquid and bubbles can also lead to the decomposition of solutes due to the high temperature reached. The violent collapse of bubbles can additionally degrade solutes by shear force. Sonication is already employed in many food applications such as emulsifying, homogenizing, sterilizing, extracting, degassing, crystallizing or inactivating microorganisms and enzymes [14]. In winemaking, US technology has already been studied in different stages of the process, namely: Yeast lysis and accelerated aging of wines [15,16], color evolution [17], vessels sanitation [18], prevention of wine spoilage by microorganisms [19], and the release of compounds located in the skin [20,21], with encouraging results, however research on this topic is still ongoing and needs to be developed.

This work aimed to verify the effect of the US treatment as a potential technology in white winemaking protocols for the extraction of sulfur aroma precursors in Sauvignon Blanc grapes. The absence of an increase of 3-S-cysteinyl mercaptohexan-1-ol (Cys-3MH) or 3-S-cysteinyl mercapto-4-methyl-pentan-2-one (Cys-4MMP) and the decrease of 3-S-glutathionyl mercaptohexan-1-ol (GSH-3MH) after ultrasound treatment, led us to verify the possibility of an interaction between sonication and thiol precursors, in order to gain further knowledge to be possibly valuable in winemaking.

## 2. Materials and Methods

### 2.1. Precursors Concentration in Marc and Juice

The concentration of aromatic thiol precursors in marc and grape juice after pressing was obtained with 13 lots of Sauvignon Blanc (SB) weighing 10 kg each, harvested on the same day from different plots in hilly areas between 190 and 510 m above sea level in Trentino (Italy). After crushing–destemming (Ares 15, OMAC s.r.l., Corridonia, MC, Italy), samples were pressed three times (5 min at 3.5 bar; 20 L Hydropress, Spiedel GmbH, Ofterdingen, Germany) at the E. Mach Foundation experimental winery (San Michele all’Adige, Italy), recording the pressing yield for each sample.

### 2.2. Ultrasound Application in Grape Must 

A 5 kg sample of SB were all collected on the same day in different plots of the same production area as above in 2017. The berries were separated from the stems, cutting the pedicel with a small pair of scissors in order to maintain the integrity of the berry and limit the *de novo* formation of precursors. After homogenization, each sample was divided into two subsamples: A 2 kg sample for the US treatment experimental design, and the remainder used for the analysis of the main quality control parameters of grape juice. In the case of the US experiment, three aliquots weighing 100 g each were manually crushed and sonicated for 3 and 5 min. Sonication was performed using an ultrasonic processor (Sonoplus HD 2200, Bandelin electronic, Berlin, Germany) with a 13 mm titanium sonotrode probe. Treatment times and amplitude applied were set according to the results of a previous work [22], adjusting the conditions to white winemaking protocols. All samples were treated using a 20 kHz frequency in continuous setting, an energy input of 153 µm amplitude, with a total nominal output of 200 W.

Samples and replicates were randomly processed and after treatment, the juice was obtained by manual pressing performed by a single person until a 60% (*v*/*w*) yield was obtained. An aliquot was used for the analysis of the main quality control parameters. A further aliquot was employed in the determination of phenol parameters and conductivity. Finally, 25 mL of juice were infused into methanol (1:1 *v*/*v*) previously chilled at −20 °C for the thiol analysis in order to limit the enzymatic reactions that lead to the *de novo* formation of thiol precursors. All samples were stored at −20 °C until analysis. The comparison between treatments was performed using the mean process values (*n* = 3) for each grape lot.

### 2.3. Ultrasound Application in Model Solution

Synthetic solution experiments were performed with a 5 L water solution supplemented with potassium chloride (3 g/L) and tartaric acid (6 g/L), adjusted to pH 3.0 with sodium hydroxide. The solution was then spiked with thiol precursors: GSH-3MH (140 µg/L), Cys-3MH (30 µg/L), GSH- 4MMP (60 µg/L), and Cys-4MMP (83 µg/L). Five samples of the solution underwent a 5 min US treatment at the same conditions of the grape must experiment (20 kHz; 153 µm), with the temperature recorded every 10 s. Five other samples were heated in a thermostatic water bath until a comparable maximum temperature in a similar amount of time was achieved. Samples thus obtained were then frozen at −20 °C until analysis. 

### 2.4. Analytical Methods

#### 2.4.1. Reagents

The ultrapure water was produced using the Arium Pro UV OF Ultrapure Water system (Sartorius, Göttingen, Germany). Formic acid and acetonitrile LC/MS grade, potassium metabisulfite ≥ 97% were purchased from Sigma-Aldrich (Milan, Italy). D3-(R/S)-3-S-cysteinylhexan-1-ol, D3-(R/S)-glutathionylhexan-1-ol and their deuterated derivatives (Cys-3MH and GSH-3MH) were purchased from Buchem BV (Apeldoorn, The Netherlands).

#### 2.4.2. LC-MS/MS Analysis of Thiol Precursors

The thiol precursors were determined using an Acquity Ultra Performance Liquid chromatographer (UPLC Waters Corporation, Milford, MA, USA) coupled with a Xevo TQ MS mass spectrometer (Waters Corporation, Milford, CT, USA) according to the method and conditions reported by Larcher et al. [6]. Five μL of sample were injected into an Acquity UPLC HSS T3 C18 chromatographic column (1.8 μM film thickness, 2.1 mm × 100 mm; Waters) with a flow rate of 0.45 mL/min and column temperature of 40 °C. Eluents A and B were water and formic acid in water at 0.1% (*v*/*v*), respectively. Gradient elution program: 5% B for 2 min, increase to 100% B in 5 min, isocratic for 1 min, decrease to 5% B in 0.01 min, finally isocratic 5% B for 2 min. The analysis was conducted in isotopic dilution operating in a positive-ion mode (capillary voltage, 2.5 kV) and with argon stream 0.20 mL/min (collisional gas) and nitrogen 1000 L/h (desolvation gas). Cone voltage potential, collision energy, and other instrumental specifications can be found in the mentioned manuscript.

#### 2.4.3. GC-MS/MS Analysis of Free Thiols

For the analysis of volatile thiols, 1-hexanethiol (0.5 mL at 0.25 mg/L) was used as internal standard, using ethyl propiolate as derivatization agent. Quantification of volatile thiols was performed using a Varian 450 gas chromatograph (Middelburg, The Netherlands) equipped with Varian 300 triple quadruple mass spectrometer (Walnut Creek, CA, USA). A 30 m × 0.25 mm × 0.25 µm DB5- MS capillary column (J&W, Agilent Technologies Italia, Milan, Italy) was used for chromatographic separation, injecting 1 µL in splitless mode (1 min) at 250 °C and using helium as carrier gas (1 mL/min). The oven temperature program was as follows: Seventy °C, held for 3.5 min, raised to 210 °C at a rate of 10 °C/min. The oven temperature was then increased to 300 °C at 15 °C/min and held at this temperature for 2 min. The mass spectrometer was operated in a multiple reaction monitoring (MRM) mode, at ionization energy of 70 eV, and using argon as the collision gas (1.8 mTorr). Further details of sampling preparation and analysis conditions have been reported by Larcher and collaborators [23].

#### 2.4.4. Spectrophotometric and Conductivity Analysis

Conductivity (mS/cm) was measured using a microCM 2202 (Crison, Carpi, Italy). Total polyphenols, hydroxycinnamates-tartaric acids, catechins, and condensed tannins were spectrophotometrically determined according to the methods reported by Singleton [24], Ribéreau-Gayon [25], Zironi [26], and Bate-Smith [27], respectively.

#### 2.4.5. Quality Control Parameters of Grape Juice

Soluble solids (°Brix), pH, volumetric density, titratable acidity, tartaric and malic acids, potassium, and yeast assimilable nitrogen (YAN) were determined using a WineScanTM FT 120 Type 77310 (Foss Electric A/S Hillerød, Denmark) calibrated according to the official methods of the International Organization of Vine and Wine [28].

## 3. Results and Discussion

### 3.1. Precursors Concentration in Marc and Juice

The chemical composition of the main parameters for quality control of juice is shown in Table 1. Samples showed the composition variability that was looked for, to give a better assessment of the industrial conditions of the production area; Trentino is an alpine region that belongs to the C-I area according to EU (Reg 1308/2013) based on its environmental and weather conditions that frequently determine the harvest date, which depends on climate features and grape soundness, rather than fruit ripeness. Table 1 also reports the concentration of thiol precursors in juice and marc. On average, grape marc showed concentrations of GSH-3MH and Cys-3MH of 1462 ± 745 and 714 ± 234 µg/kg, respectively, values that are significantly higher (Fisher LSD test; *n* = 13; *p* < 0.05) than those found in juice: 116 ± 37 µg/kg of GSH-3MH and 43 ± 19 µg/kg of Cys-3MH. For both, the glutathionylated precursor of 3MH was present in higher amounts than the cysteinylated one in every sample.

The concentration of 3MH precursors in juices is in accordance with data previously reported for SB [5,29] and for both juice and marc, such concentration is similar to that reported in previous studies where samples came from the same area of cultivation and were obtained with a similar procedure [9,23,30]. The pressing yield ranged between 60.4% and 67.8% of must (*w*/*w*) and made it possible to determine the total content of 3MH precursors in each fraction. 88% of the precursors were located in marc, a proportion similar to that reported between the skins and the pulp of SB grapes [31], notwithstanding a different sampling procedure and the extraction exerted during pressing. Along with the variability reported in grapes, a de novo formation of 3MH precursors has also been reported during grape handling [4,7,32], that could have affected the concentration in grape fractions and thus the proportion.

At the conditions of the experiment, a huge proportion of the aroma potential remained unused in marc and could be consequently valuable for winemaking purposes. SB grapes undergo prefermentation maceration in order to maximize the extraction of aroma compounds, including the precursors of the varietal thiols, molecules that characterize the aroma of its wines [33]. However, this step could be harmful due to the risk of development of spoilage microorganisms or of uncontrolled oxidation processes. For this reason, we wanted to investigate whether US could be useful in speeding up and enhancing the extraction of thiol precursors from white grape skins, based on the results reported by Ferraretto and Celotti [22] who applied this technology in red winemaking.

### 3.2. Ultrasound Application on Grape Must

As for the former experiment, samples were collected on the same day to ensure a reasonable ripeness variability and the complete soundness of grapes. US is a physical treatment that acts by degrading the cell wall of the skin [20] where most of the precursors are located and should work regardless of ripeness. The required variability prerequisite was satisfied and confirmed by the analysis of the maturity parameters: pH ranging between 2.92 and 3.09, soluble solids between 17.9 and 22.1 °Brix, variation in potassium values between 1.39 and 1.47 g/L, and YAN between 129 mg/L and 211 mg/L. With regards to the acidity parameters, titratable acidity ranged between 8.6 and 10.2 g/L, tartaric acid between 6.4 and 8.4 g/L, and malic acid between 4.4 and 5.8 g/L. All values were in accordance with the former dataset, and four out of the five samples exceeded 12% of potential ethanol, which is considered to be the industrial ripeness target in this production area. The values of pH, malic acid, and total acidity could be acceptable for winemaking of Sauvignon Blanc in this environment, with the characteristic altitude of vineyards and climatic area. It seems, however, that the grapes could have ripened further. Ripeness is irrelevant for the purpose of the experiment, even though a premature harvest could have prevented the second requirement from being satisfied. Indeed, it has been reported that the presence of Botrytis cinerea in grapes enhances thiol precursors concentration [34] and degrades skin cells through the pool of enzymes secreted by this mould [35], thus its absence was essential for a proper evaluation of the effectiveness of the US treatment.

The mean values of conductivity and the concentration of catechins, tannins, total phenols, hydroxycinnamate-tartaric acid, and thiol precursors are shown in Table 2. Conductivity and phenol parameters were used as extraction indicators [24,25,26,27], thus making it possible to determine the efficacy of the treatment on cell wall breakdown. The evaluation of the effect was performed applying the Fisher’s least significance difference test using the mean process values for each grape lot. Every extraction indicator presented an upward trend with the US treatment duration applied. These differences were significant after 5 min of sonication against control samples for conductivity, catechins and total polyphenols (*p* < 0.05). The lack of significance in tannin content could be justified by the fact that most of the tannins in grapes are found in seeds and extraction from this tissue during vinification is commonly accompanied by the presence of ethanol [36]. In turn, hydroxycinnamate tartaric acids undergo enzymatic reactions, being considered one of the most reactive compounds in grape must [37]. Although concentration of these acids increases over time, the results are the combination of several mechanisms that would have led to the lack of significance. Nevertheless, the positive trend for all the parameters and the significant increase of conductivity, catechins, and total phenols would confirm the efficacy of the treatment in breaking down different berry tissues as previously reported for phenols during maceration of sonicated grapes in red winemaking [20,22].

**Figure 2:**
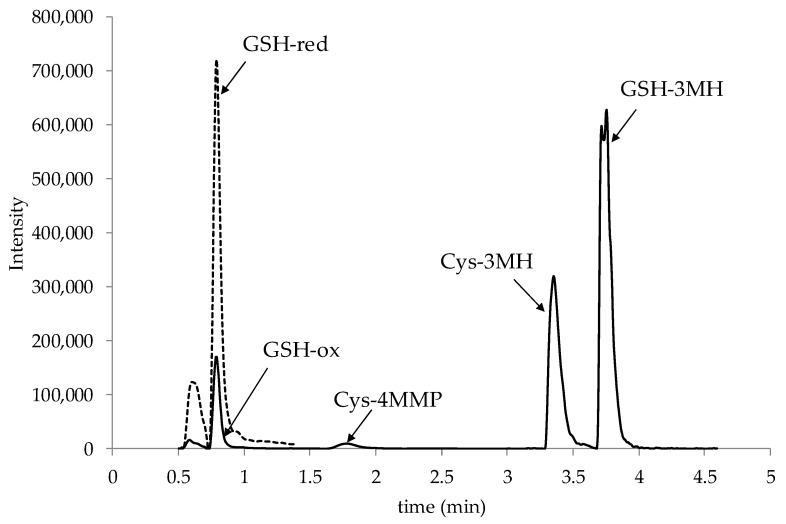
Extract ion chromatogram of must grape recorded in Multiple Reaction Monitoring (MRM) mode.

Regarding sulfur compounds, no sample—treated or not—showed values of GSH-4MMP above the limit of detection (1 µg/L). Sonication did not cause significant differences in the concentration of any of the thiol precursors and GSH-3MH showed a surprising negative trend unlike the results reported for conductivity, catechins, and total phenols. The trend was reported in every grape lot and was unexpected considering the richness of thiol precursors in the marc of the former experiment. This suggests different scenarios. One is linked to the inactivation of some of the enzymes involved in the biogenic pathway of the de novo formation of precursors by means of the sonication treatment, already reported for other enzymes [38]. New precursors can be formed under oxidizing conditions by means of the hexen-2-al originated from lipids by grape endogenous lipoxygenases. Hexen-1-al in turn can then react with GSH or Cys to form new adducts [39]. Hence, the enzyme inactivation could limit the de novo formation if compared to controls. The adopted analytical method made it possible to detect (even though not to quantify) GSH—a known endogenous antioxidant of grapes that undergoes oxidation reactions in grape must [37]—and GSSG, an oxidized form of GSH (Figure 2).

The analysis of the peak area against the area of the internal standard used showed that every treatment had a different GSSG/IS ratio (Fisher’s LSD test; *n* = 5; *p* < 0.05), showing a negative trend with the duration of the treatment (0.106 ± 0.021, 0.084 ± 0.015, and 0.071 ± 0.014, respectively for control, 3 and 5 min). The reactivity of GSH in grape juice is preferentially linked to its ability to reduce o–quinones formed following the enzymatic oxidation of hydroxycinnamates-tartaric acid by means of polyphenol oxidases [37]. The lower content in GSSG/IS in treated samples would have followed along the same direction of the enzymatic inhibition caused by US. However, the undifferentiated values of the GSH/IS ratio between treatments (0.276 ± 0.336, 0.280 ± 0.623, and 0.276 ± 0.306, respectively for control, 3 and 5 min) would suggest the possibility of an interaction between US and thiol precursors as a second scenario: The breakage of the precursors following the treatment. It is known that sonication provokes the formation of cavitation bubbles in the liquid and their violent collapse creates high temperature and high pressure that can chemically interact with molecules. This interaction is due to ionization mechanisms, forming radicals within bubbles that can also participate in redox reactions in the interfacial shell between bubbles and liquid media [40]. To the best of our knowledge, there are no specific studies regarding the effect of sonication on thiol precursors. However, the breakage of thioethers [41] or peptides [42] following sonication is reported. Thus, the divergence of the results regarding the extraction, the decrease of GSSG/IS ratio, and the lack of significance on the GSH/IS ratio values after US treatment could suggest that the –S– bond may undergo a cleavage reaction. The evaluation of the degradation of thiol precursors at the conditions of the experiment cannot be conclusive, as the results are the combination of the extraction mechanisms, the de novo formation of precursors and, eventually, the breakage of the molecules. Previous studies reported that the application of ultrasound in grapes determines an increase of the free thiols in Sauvignon Blanc wines [43], assuming that this could possibly be the result of the increase in the extraction of precursors from skins, which is in contrast to what we found in our study. Thiols can be formed sonochemically among the degradation products of thioeters after the cleavage of the C–S or S–S bounds and the subsequent combination of the free radicals [41]. This possibility is particularly interesting if the hypothesis of the degradation of precursors would lead to the formation of the aromatic molecules from the nonvolatile compounds. The analysis of 3MH and 4MMP in juices after sonication would have not, however, give a certain explanation. Both compounds have been reported to react with catechins [44,45], which concentration is, inter alia, enhanced by treatment. Moreover, the degradation of the sulfhydryl groups of the compounds formed cannot be excluded. The evaluation at the end of the alcoholic fermentation could also be inconclusive and the results would be the accumulation of a number of factors that affect the pathways that lead to the liberation and de novo formation of thiols or their preservation during aging [46]. To examine if the lack of a positive effect on the extraction of thiol precursors could be a result of their interaction with ultrasound, and eventually if the degradation leads to the formation of the aroma molecules, an experiment was performed in a model solution, mimicking the matrix conditions of grape juice.

### 3.3. Ultrasound Application in Model Solution

Treatment with US in the model solution was performed for 5 min in the same conditions as above. During this time, the temperature was recorded every 10 s in order to determine the maximum reached and the rate, thus permitting the parameters of the thermostatic bath for the preparation of the control samples to be set up properly. Temperatures increased constantly in sonicated samples with a first order rate [ΔT (°C) = 0.115·t (s)] at ambient conditions, showing a good correlation in the range under study (*R*^2^ = 0.999) and reaching a maximum variation of 33 °C. Control samples were then heated up to the same temperature in a similar amount of time in order to separate the effect of temperature from sonication.

**Figure 3:**
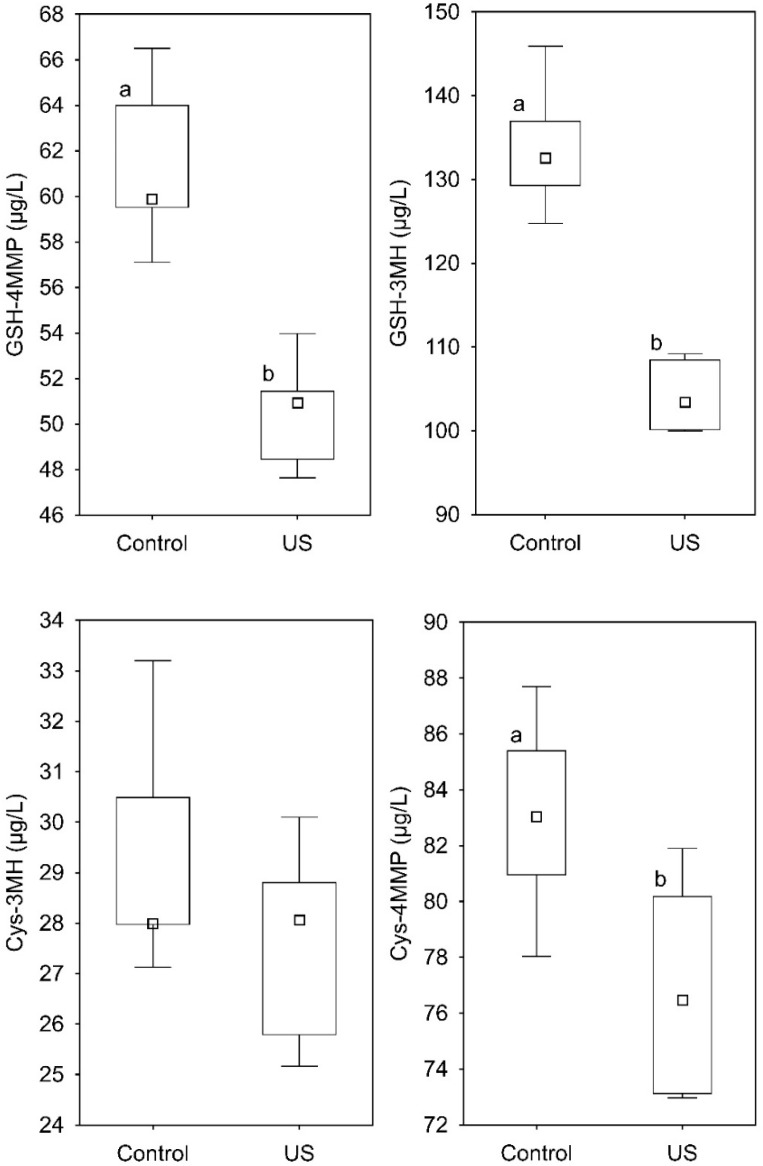
Concentration distribution of 3-S-glutathionyl mercaptohexan-1-ol (GSH-3MH), 3-S-cysteinyl mercaptohexan-1-ol (Cys-3MH), 3-S-glutathionyl mercapto-4-methyl-pentan-2-one (GSH-4MMP), and 3-S-cysteinyl mercapto-4-methyl-pentan-2-one (Cys-4MMP) in the model solution of control and treated (US) samples after 5 min of sonication. Different letters indicate mean values statistically differentiated.

The results distribution, split by treatment and precursor, are shown in the box plots of Figure 3. The mean concentrations (*n* = 5) of GSH-3MH, GSH-4MMP, Cys-3MH, and Cys-4MMP in control samples was 133 ± 7, 62 ± 4, 29 ± 2, and 84 ± 5 µg/L, respectively, and 104 ± 4, 50 ± 3, 28 ± 2, and 77 ± 4 µg/L after the US treatment. The analysis of the variance (Fisher LSD test; *p* < 0.05) found significant differences between treatments with regards to GSH-3MH, GSH-4MMP, and Cys-4MMP, confirming the interaction of US and thiol precursors. The reduction of the concentration was higher on average on glutathionylated than on cysteinylated forms: ~−22.4% and ~−18.0% for GSH-3MH and GSH-4MMP, respectively and~−5.6% and ~−7.6% for Cys-3MH and Cys-4MMP. This situation is in accordance with the results found in grapes, where the concentration of GSH-3MH was the one that was found to be reduced after treatment. The breakage of the compounds would be possibly related to the radical and electrontransfer processes generated by the high energy and excited state species created in the solution following cavitation. The mechanism of the cleavage and the degradation products do not make part of the present work and specific studies should be performed. Nevertheless, from a sensory point of view, precursors are key molecules in the aroma of certain wines, insofar as volatile thiols released during fermentation present extremely low odor thresholds. Thus, in case the precursor cleavage occurs, the release of 3MH and 4MMP should represent the most important reaction products in winemaking.

**Figure 4:**
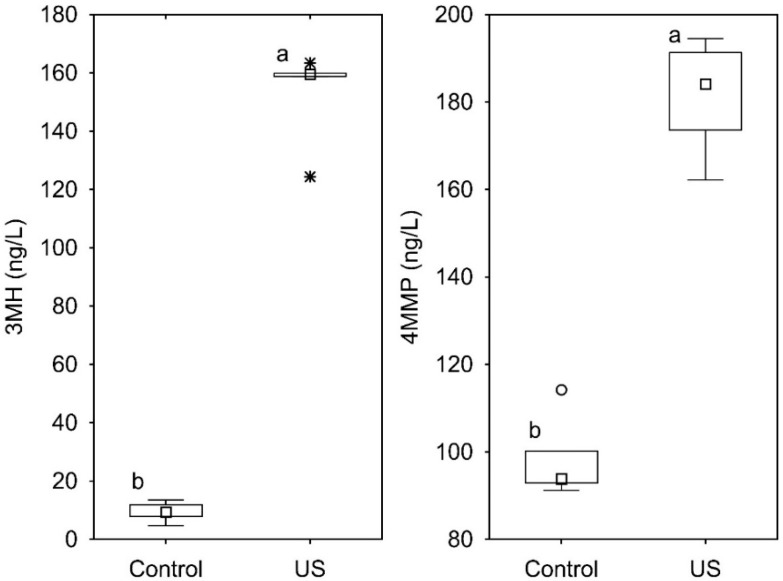
Concentration distribution of 3-mercaptohexan-1-ol (3MH) and 4-mercapto-4-methyl pentan-2-one (4MMP) in the model solution in control and treated (US) samples after 5 min of sonication. Different letters indicate mean values statistically differentiated. * Outliers.

After sonication, the mean concentration of 3MH and 4MMP was 147 ± 19 and 177 ± 15 ng/L, respectively, values significantly higher than those of the control samples: 9 ± 3 and 96 ± 12 ng/L (Fisher LSD test; *n* = 5; *p* < 0.05). The distribution of the results of the five replicates is shown in Figure 4. The degradation of the precursors fitted then with an increase of 3MH and 4MMP and in the experimental conditions, the sonicated samples showed on average 138 ng/L of 3MH and 81 ng/L of 4MMP more than the untreated samples. On a molar basis, the release ratio of 3MH and 4MMP due to treatment, with respect to the net decrease of each precursor is 1.3% for 3MH and 1.1% for 4MMP. A quantitative analysis of the results should not, however, be taken into consideration, not only because of the possibility of degradation of the volatile thiols within the cavitation bubbles or following the formation of radicals, but also due to the stripping effect caused by bubble collapse and the high volatility of thiols, which is enhanced by temperature.

It is necessary to further investigate this phenomenon to be exploited in winemaking. On the one hand, the mechanisms of interaction between US and thiol precursors should be identified. If the formation of radicals is involved, this would be strongly dependent on the process variables (e.g., intensity or time applied) and on the matrix composition. Among the last, low values of pH would favour the formation of radicals [47] and then promote the mechanism. On the other hand, the degradation products of the reaction should be further studied. Verifying the formation of the aroma molecules at oenological conditions would be particularly interesting for winemakers. Nowadays, the ratio between volatile thiols in wine and grape precursors in musts is very low [48] and the biogenic origin of the aroma molecules present in wines from precursors is only explicable for a 50% [49]. Consequently, US would allow enhancing the tropical scents of wines through specific vinification protocols.

## 4. Conclusions

The positive effect of ultrasound on the extraction mechanisms in grape must was confirmed by the increase of conductivity and phenols. This effect was not however detected in any of the sulfur aroma precursors studied, and the concentration of 3-mercaptohexan-1-ol linked to glutathione even showed a negative trend. This divergence on the effects of ultrasound enabled us to study the possible interaction between ultrasound and thiol precursors. The study was performed in synthetic media to avoid the interference of phenols and the effect of ultrasound in sulfhydryl groups. The concentration of GSH-3MH, GSH-4MMP, and Cys-4MMP significantly decreased after treatment in synthetic solution, more evident for those thiols linked for glutathione, as reported in grape juice. The diminution of the precursor concentration was accompanied by an increase of 3-mercaptohexan-1-ol and 4-mercapto-4-methyl pentan-2-one, suggesting that the volatile molecules could be enhanced by US. 

Further research is needed to investigate the mechanisms of interaction between ultrasound and thiol precursors and the potential degradation products during winemaking conditions. This could allow in depth examination on how to exploit these findings in winemaking of aromatic cultivars, not only based on thiol aroma but for the overall quality of the final product.

## Figures and Tables

**Table 1:** Quality control parameters and concentration of thiol precursors of Sauvignon Blanc grape samples (*n* = 13) of the semi-industrial trial.

Grape Sample	°Brix	pH	Titratable Acidity (g/L)	Tartaric Acid (g/L)	Malic Acid (g/L)	Potassium (mg/L)	YAN (mg/L)	GSH-3MH (μg/kg)		Cys-3MH (μg/kg)	
								Juice	Marc	Juice	Marc
A	21.9	3.13	5.8	4.0	2.8	795	168	121	802	19	377
B	23.2	2.81	10.9	9.2	3.1	1581	<20	57	486	29	534
C	23.6	2.82	10.5	8.6	3.5	1552	<20	95	436	33	446
D	22.8	2.85	9.3	8.3	2.6	1386	41	84	1555	32	799
E	22.7	2.81	9.7	8.9	2.4	1479	24	100	1055	32	842
F	22.2	2.83	9.2	8.3	2.6	1352	23	122	2388	31	807
G	21.5	2.86	10.0	8.4	3.8	1417	120	120	2049	30	1175
H	20.1	2.88	10.4	8.7	3.9	1476	148	208	2233	50	953
I	21.2	2.97	9.6	8.3	3.9	1490	147	120	932	28	865
J	19.9	2.88	10.2	7.7	4.1	1134	131	129	2306	70	842
K	21.8	2.94	9.5	7.4	3.9	1181	144	155	2478	79	641
L	18.7	2.82	10.3	7.9	3.6	1126	90	106	1130	57	500
M	20.6	2.81	10.1	8.0	3.3	1114	60	92	1179	64	503

YAN: Yeast assimilable nitrogen; GSH-3MH: 3-S-glutathionyl mercaptohexan-1-ol; Cys-3MH: 3-S-cysteinyl mercaptohexan-1-ol.

**Table 2:** Mean values and standard deviations of Sauvignon Blanc grape samples of the ultrasound lab-scale experiment.

Parameter	Control (*n* = 5)		3 min (*n* = 5)		5 min (*n* = 5)	
Conductibility (mS/cm)	2.06 ± 0.22	b	2.41 ± 0.26	a	2.61 ± 0.22	a
Catechins (mg/L)	8.4 ± 2.0	b	18.2 ± 5.1	b	37.4 ± 20.8	a
Tannins (mg/L)	17.69 ± 4.11		20.70 ± 4.31		21.96 ± 3.97	
Total polyphenols (mg/L)	403.2 ± 44.7	b	510.1 ± 134.5	ab	624.5 ± 160.9	a
Hydroxycinnamate-tartaric acids (mg/L)	1.61 ± 0.35		1.82 ± 0.52		2.12 ± 0.67	
GSH-3MH (µg/L)	168 ± 43		156 ± 36		149 ± 32	
Cys-3MH (µg/L)	96 ± 44		95 ± 44		97 ± 41	
Cys-4MMP (µg/L)	13 ± 3		12 ± 3		12 ± 3	

Different letters indicate values statistically differentiated with the Fisher’s Least Significant Difference test (*n* = 5; *p* < 0.05). (GSH-3MH: 3-S-glutathionyl mercaptohexan-1-ol; Cys-3MH: 3-S-cysteinyl mercaptohexan-1-ol; Cys-4MMP: 3-S-cysteinyl mercapto-4-methyl-pentan-2-one).
